# How to communicate evidence against catchy erroneous arguments?

**DOI:** 10.1093/eurpub/ckag003

**Published:** 2026-01-20

**Authors:** Avi Magid, Dorit Nitzan, Charlotte Marchandise, Ricardo Mexia, Emilia Aragon de Leon, Alison McCallum

**Affiliations:** Department of International Health, Maastricht University, Maastricht, The Netherlands; Association of Schools of Public Health in the European Region (ASPHER), Brussels, Belgium; School of Public Health, Ben Gurion University of the Negev, Beer-Sheva, Israel; EUPHA, Utrecht, The Netherlands; Epidemiology Department, National Institute of Health Doctor Ricardo Jorge, Lisbon, Portugal; Division of Country Health Policies and Systems, WHO Regional Office for Europe, Copenhagen, Denmark; Association of Schools of Public Health in the European Region (ASPHER), Brussels, Belgium; Centre for Population Health Sciences, University of Edinburgh, Edinburgh, Scotland

In a workshop at the European Public Health conference 2025, we deployed forum theatre alongside traditional presentations to examine our ability as public health professionals to communicate evidence informed, practical messages that foreground protecting public health and to convince a public health audience to support them. Participants were asked to intervene as participators in a staged debate between pro-WHO and anti-WHO politicians in a fictional parliament. The pro-WHO side used evidence-based arguments, whereas the anti-WHO side used misinformation and populist arguments, claiming that WHO is distant, politicised, unaccountable, hides information from ‘ordinary people’, suggesting that national sovereignty should replace global cooperation and multilateral governance. These inaccurate arguments were deliberately framed to evoke identity, frustration, and distrust—features known to increase persuasive power and limit scrutiny of the underpinning arguments [1].

The results were striking. During the workshop, we observed that it was easier to follow and support the anti-WHO side even for an audience of public health professionals. The first audience vote favoured withdrawing support for WHO. Many participants initially found the emotionally based, anti-evidence narrative more compelling than evidence-based arguments. Reactions in the room—laughter, nodding, murmurs of agreement—suggested alignment with the dissenting side. We observed that even those committed to protecting public health struggled to translate evidence into persuasive messages. This experiment exposed a critical problem: misinformation spreads easily, and populist disinformation is easier to promulgate than careful, evidence-based messages, even for expert communicators. In a world where anything can be said, regardless of its truth, we struggle with limited resources against a well-resourced the other non-evidence-based side tactics. Our findings are supported by others, demonstrating the speed and efficiency with which misinformation spreads. Vosoughi and colleagues, examining over 126 000 Twitter cascades, found that falsehoods reach more people and diffuse faster than truths, driven by novelty, emotional arousal, and cognitive biases [2]. Misinformation exploits heuristics, identity, and motivated reasoning, making it resistant to correction even among highly educated individuals [1]. Systematic reviews confirm that misinformation is widespread and highly engaging across platforms [3].

Our workshop brought the literature to life through the experience of a microcosm of the public health workforce. The anti-WHO narrative succeeded because it adopted the features that make misinformation effective: simplicity, emotional appeal, and alignment with broader cultural narratives. By contrast, the pro-WHO arguments tended to be technical, institutional, or abstract—accurate but less resonant. This experience demonstrated that our communication remains structured around evidence, not persuasion.

We assume that facts speak for themselves. They do not. This matters because WHO—and global health more broadly—is increasingly targeted and attacked by politicised narratives and bad faith actors. Claims that WHO acts as an overreaching global authority, restricts national autonomy, or operates under hidden influence have increased across political movements in the US, in Europe and beyond. These narratives exploit inequity, frustration, identity, and distrust, the root causes of which are the same actors promoting weak and anti-WHO rhetoric, undermining international cooperation at a time when global threats—from pandemics to climate change—require the opposite. If public health professionals struggle to advocate for public health among themselves, we must change our relationships with policymakers and communities. To respond effectively, we must confront an uncomfortable reality: despite our perception of ourselves as advocates, public health communication has long relied on evidence summaries, expert consensus, and appeals to global solidarity. These remain essential, but insufficient in our polarized world. Research on persuasive health communication shows that framing is important [4], narrative strategies increase comprehension and reduce resistance, while prebunking techniques build resilience against manipulation [4]. Values-based framing—linking health messages to widely held principles such as fairness, security, or autonomy—can increase trust and engagement [5]. Yet these approaches are not systematically embedded in public health practice. The workshop highlighted the need to reframe our messages. Appeals to global cooperation or “health for all,” may be central to public health identity, but resonate less with our audiences. Messages grounded in personal relevance, national interest, or community well-being may be more effective. This does not require abandoning global values but articulating them in ways that connect with the concerns shaping public opinion.

We must strengthen communication capacities within the public health workforce. If we expect professionals to counter misinformation, we need to prepare them to do so. This includes training in framing, narrative construction, rapid response, debating skills, and the factors underpinning misinformation [4]. WHO’s own infodemic management agenda underscores the need for tools, methodologies, and workforce competencies that match the speed and scale of misinformation [6]. Many of these capacities remain underdeveloped. Finally, it is important to recognise that defending WHO is not a one-time task but part of a broader effort to safeguard trust in global cooperation and non-partisan multilateral governance. When misinformation undermines the WHO, it risks the smooth running of our local health systems, that rely on the international classification of disease and death, medicines and technologies and global professional standards. It also weakens pandemic preparedness, disrupts coordinated responses to cross-border incidents, and fuels political narratives that prioritise isolation over solidarity. Our workshop provided situational awareness and lessons for the future. Public health professionals are committed, knowledgeable, and deeply invested in evidence-based practice. Yet, when confronted with misinformation’s tactics, we are too often out communicated. The threat is not only the content of misinformation but its methods: emotional resonance, simplicity, and strategic framing. To counter this, we need to re-evaluate our approaches, be innovative and consider alternative tactics. We call on the wider public health community to help us re-think how we present evidence, integrate behavioural and political science into our communications, engage with values and adopt visual and narrative approaches, to get our messages across.

Conflict of interest: The authors declare that there are no conflicts of interest.

## Data Availability

No new data were generated or analysed in support of this editorial.
